# Foliar nutrient supplementation with micronutrient-embedded fertilizer increases biofortification, soil biological activity and productivity of eggplant

**DOI:** 10.1038/s41598-022-09247-0

**Published:** 2022-03-25

**Authors:** Ram Swaroop Bana, Gograj Singh Jat, Minakshi Grover, Shanti D. Bamboriya, Deepak Singh, Ruchi Bansal, Anil K. Choudhary, Vipin Kumar, Alison M. Laing, Samarth Godara, Ramesh Chand Bana, Hement Kumar, Bhola Ram Kuri, Achchhelal Yadav, Teekam Singh

**Affiliations:** 1 Division of Agronomy, ICAR-Indian Agricultural Research Institute, New Delhi, 110 012 India; 2 Division of Vegetable Science, ICAR-Indian Agricultural Research Institute, New Delhi, 110 012 India; 3 Division of Microbiology, ICAR-Indian Agricultural Research Institute, New Delhi, 110 012 India; 4 ICAR-Indian Agricultural Statistics Research Institute, New Delhi, 110 012 India; 5 ICAR-National Bureau of Plant Genetic Resources, New Delhi, 110 012 India; 6 ICAR-Central Potato Research Institute, Shimla, 171 001 India; 7 Eastern Shore Agricultural Research and Extension Center, Virginia Tech, Painter, VA 23420 USA; 8 CSIRO Agriculture & Food, St. Lucia, Brisbane, 4067 Australia; 9 Division of Agricultural Physics, ICAR-Indian Agricultural Research Institute, New Delhi, 110 012 India

**Keywords:** Microbiology, Plant sciences

## Abstract

Micronutrient malnutrition or hidden hunger remains a major global challenge for human health and wellness. The problem results from soil micro- and macro-nutrient deficiencies combined with imbalanced fertilizer use. Micronutrient-embedded NPK (MNENPK) complex fertilizers have been developed to overcome the macro- and micro-element deficiencies to enhance the yield and nutritive value of key crop products. We investigated the effect of foliar applications of an MNENPK fertilizer containing N, P, K, Fe, Zn and B in combination with traditional basal NPK fertilizers in terms of eggplant yield, fruit nutritive quality and on soil biological properties. Applying a multi-element foliar fertilizer improved the nutritional quality of eggplant fruit, with a significant increases in the concentration of Fe (+ 26%), Zn (+ 34%), K (+ 6%), Cu (+ 24%), and Mn (+ 27%), all of which are essential for human health. Increasing supply of essential micronutrients during the plant reproductive stages increased fruit yield, as a result of improved yield parameters. The positive effect of foliar fertilizing with MNENPK on soil biological parameters (soil microbial biomass carbon, dehydrogenase, alkaline phosphatase) also demonstrated its capacity to enhance soil fertility. This study suggests that foliar fertilizing with a multi-nutrient product such as MNENPK at eggplant flowering and fruiting stages, combined with the recommended-doses of NPK fertilizers is the optimal strategy to improve the nutritional quality of eggplant fruits and increase crop yields, both of which will contribute to reduce micronutrient malnutrition and hunger globally.

## Introduction

Eggplant (*Solanum melongena L.*) or brinjal is the fifth most important vegetable crop globally^1,2^, grown on approximately 1.86 million hectares of croplands, with annual global production of ⁓54 million tonnes worth > USD 10 billion^2^. While eggplant is produced across Asia and Europe and in many African nations^3^, of the total global eggplant production China and India are the major producers, generating 61% and 23%, respectively, of the total annual yield^2^. The average eggplant-fruit yield in South and East Asia is far below the potential productivity, primarily as a result of abiotic and biotic plant stresses and poor awareness of the correct nutrition and other management practices among farmers^4^. Eggplant is a high nutrient-exhausting crop, as a result of its high biomass production and long-growing season. On an average, for each tonnes production, the eggplant crop removes 3.16, 0.18, 2.13 kg ha^−1^ nitrogen (N), phosphorus (P), and potassium (K) respectively, along with significant amounts of micronutrients from the soil^5^. Most eggplant farmers apply only primary-nutrient fertilizers which lead to low and variable crop yield and sub-optimal nutrient concentrations in the fruit. The ongoing use of primary-nutrient fertilizers combined with limited use of organic or micronutrient fertilizers has led to emergence of multi-nutrient deficiencies in the majority of soils in the Indian subcontinent ^6,7^. Across India, approximately 89, 80 and 50% of the arable soils are deficient in N, P and K, respectively. Further, zinc (Zn), boron (B), iron (Fe), manganese (Mn), molybdenum (Mo), and copper (Cu) deficiencies have been reported in around 40, 33, 12, 5, 11 and 3% of Indian soils, respectively^7–9^. Similarly, up to 51% of the arable soils in China are deficient in Zn, with deficiencies in Mo, N, Mn, Cu and Fe in 47, 34.5, 21, 7 and 5% of farmland soils, respectively, with large macronutrient (i.e. N, P, K) deficiencies also^10^. Considering the widespread multi-nutrient deficiencies in soils on which eggplants are grown, efficient nutrient management strategies to overcome these deficiencies are vitally important^7^.

Modern hybrids and high-yielding eggplant varieties are more responsive to applied fertilizers than traditional varieties. Such varieties have high production potential; they require regular fertilization during both vegetative and reproductive stages^11^. Water-soluble multi-nutrient fertilizers could provide sufficient nutrients to the plant throughout its growing season and could reduce flower and fruit drop, thus improving crop yield and quality^12^.

Additionally, crop cultivation in nutrient-deficient soils results in foods with low nutrient concentrations, particularly of micronutrients, which contributes to malnutrition and hidden hunger in many emerging-economy countries^13,14^. Globally, micronutrient malnutrition, arising either from inadequate consumption of fruits and vegetables or from consumption of foods which are low or deficient in essential micronutrients, results in approximately 1.7 million deaths annually^7,15,16^. Eating foods which have been biofortified to increase their micronutrient content is a useful and socially and economically affordable pathway to overcome malnutrition^7,13^.

In order to reduce malnutrition it is imperative to supply crops with micronutrients in addition to the macronutrients required for plant growth. The efficiency of inorganic micronutrients applied into soils is low as they become easily fixed to soil particles^17^. Applying a foliar spray of micronutrient fertilizers is an effective option to enhance plant nutrient-use efficiency (NUE)^12,18^. The recent innovation of micronutrient-embedded NPK fertilizers (MNENPK) enables growers to cater to the specific multi-micronutrient demands of individual crops at specific plant growth stages. Until now, only single- or two-nutrient foliar fertilizers have been tested for their efficacy in eggplant biofortification and yield improvement. As well, there have been no systematic field trials conducted on foliar applications of MNENPK fertilizer in eggplant crop and the subsequent effects on soil health and enzyme activity, and there is little information available on partitioning of key macro- and micro-nutrients when fertilized with MNENPK in different plant parts. The current investigation was conducted to (1) test the effectiveness of MNENPK fertilizers on eggplant growth and yield, (2) quantify the nutrient biofortification of different eggplant parts (including fruit) under MNENPK fertilizers, and (3) document soil biological activity under diverse fertility scenarios, in a south Asian semi-arid agro-ecology.

## Results

### Plant and fruit growth parameters and fruit yield

**Figure 1:**
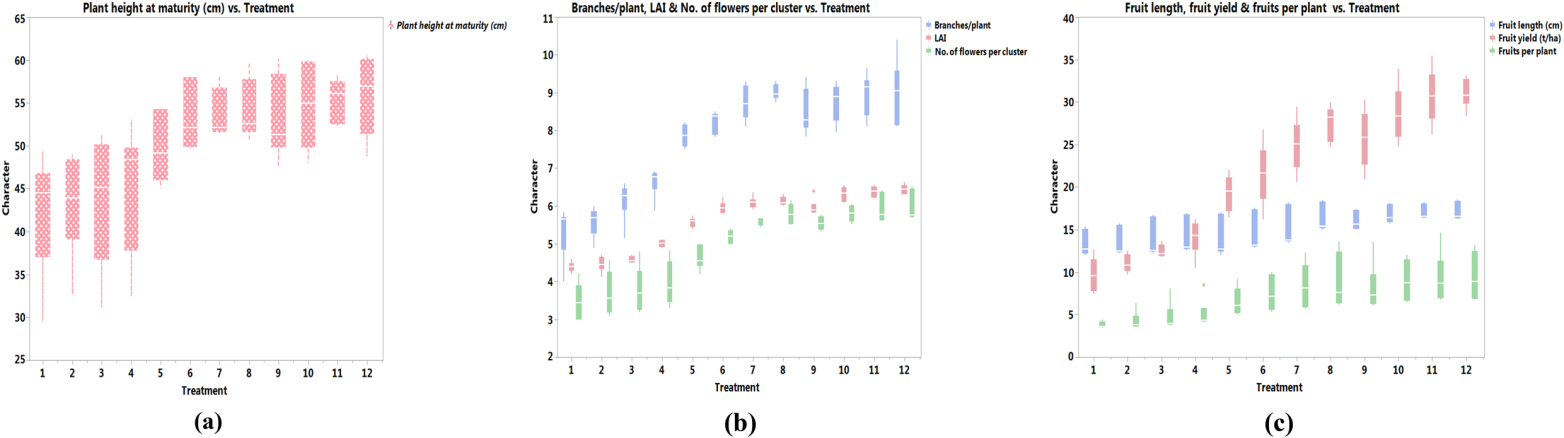
Effect of diverse fertility scenarios and MNENPK fertilizer treatments on (**a**) plant height, (**b**) growth parameters and (**c**) yield attributes and yield of eggplant (pooled data of 2017–18 and 2018–19 cropping seasons). #1 = RDF1-MNENPK1; 2 = RDF1-MNENPK2; 3 = RDF1-MNENPK3; 4 = RDF1-MNENPK4; 5 = RDF2-MNENPK1; 6 = RDF2-MNENPK2; 7 = RDF2-MNENPK3; 8 = RDF2-MNENPK4; 9 = RDF3-MNENPK1; 10 = RDF3-MNENPK2; 11 = RDF3-MNENPK3; 12 = RDF3-MNENPK4.

Of the main-plot NPK fertilizer treatments, RDF3 produced the tallest plants (54.8 cm) with the highest number of branches per plant (8.8), LAI (6.3), number of flowers per cluster (5.8), fruit length (16.7 cm), number of fruits per plant (9) and fruit yield (29.0 t/ha). All parameters were significantly higher under RDF3 treatment over RDF2 and RDF1 treatments (Fig. 1a–c). Plant growth and fruit yield were lowest under RDF1, with plant height of 43.5 cm, number of branches per plant at 5.9, LAI at 4.6, number of flowers per cluster at 3.7, fruit length at 13.7 cm, number of fruits per plant at 4.46 and fruit yield at 11.81 t ha^−1^.

The treatment MNEPK1 had the lowest values for all plant and fruit growth parameters, and fruit yield (Fig. 1a–c).

There were no statistical differences (p > 0.05) between treatments MNEPK1 and MNEPK2 in terms of plant height (48.3 and 50.4 cm, respectively), number of branches per plant (7.22 and 7.52, respectively) and number of fruits per plant (6.17 and 6.91, respectively). These later two treatments were significantly (p < 0.05) different in terms of LAI (5.31 and 5.58, respectively), number of flowers per cluster (4.55 and 4.89, respectively), fruit length (14.43 and 14.91 cm, respectively) and fruit yield (18.21 and 20.40 t/ha, respectively). Treatment MNENPK4 had the greatest plant height (51.7 cm), number of branches per plant (8.22), LAI (5.85), number of flowers per cluster (5.24), fruit length (15.83 cm), number of fruits per plant (7.83) and fruit yield (24.19 t ha^−1^), however treatment MNENPK4 did not differ statistically (p > 0.05) from treatment MNENPK3 in terms of all growth parameters except LAI and fruit length.

The interactions of RDF × MNENPK highlights that the highest plant height, number of branches per plant, LAI, number of flowers per cluster, fruit length, number of fruits per plant and fruit yield were observed in the RDF3-MNENPK4 treatment combination; although there was no statistical difference between this and the RDF3-MNENPK3 treatment. In some of the growth and yield parameters, RDF2-MNENPK4 also performed statistically at par to RDF3-MNENPK4, but all the MNENPK in combination with RDF1 showed poor growth and lower yield levels. This highlighted that enhancement of the multi-nutrient fertilizer dose from the MNENPK1 treatment (No MNENPK application) to the MNENPK4 treatment (0.75 kg ha^−1^ MNENPK), without application of recommended NPK fertilizer did not result in significant growth and yield augmentation in eggplant. Contrarily, MNENPK application in combination with either RDF2 or RDF3 did result in better growth and significant yield improvement.

### Soil fertility status

Plant-available NPK in the RDF1 treatment were 141.3, 12.1 and 206.3 kg ha^−1^, respectively; this increased in the RDF2 (171.1, 13.3 and 237.5 kg ha^−1^, respectively), and the RDF3 (182.2, 13.9 and 259.2 kg ha^−1^, respectively) treatments. There was no significant difference between the MNENPK foliar-supplementation treatments in terms of plant-available N or K, however the MNENPK_3_ (14.2 kg ha^−1^) and MNENPK_4_ (14.6 kg ha^−1^) treatments were higher in plant-available P than the MNENPK_1_ (12.9 kg ha^−1^) treatment (Table 1). Interaction effect of RDF and MNENPK foliar application were statistically non-significant in terms of available NPK content in the soil after two eggplant crops.

**Table 1:** Effect of diverse fertility scenarios and MNENPK treatments on soil after two eggplant crops.

Treatment	Available N (kg ha^−1^)	Available P (kg ha^−1^)	Available K (kg ha^−1^)
**Diverse fertility scenario**			
RDF_1_	141.3_c_	12.1_c_	202.3_c_
RDF_2_	175.1_b_	13.8_b_	238.8_b_
RDF_3_	182.2_a_	15.5_c_	263.2_a_
CD (*p* = 0.05)	9.88	1.21	21.6
**Micronutrient embedded NPK (MNENPK) fertilizer**			
MNENPK_1_	164.6_a_	12.9_c_	224.1_a_
MNENPK_2_	159.6_a_	13.6_bc_	233.7_a_
MNENPK_3_	170.1_a_	14.2_ab_	239.3_a_
MNENPK_4_	167.0_a_	14.6_a_	242.2_a_
LSD (*p* = 0.05)	NS	0.97	NS
Interaction RDF × MNENPK	NS	NS	NS

Means followed by a similar lowercase letter within a column are not significantly different at p < 0.05 according to Tukey’s HSD test; NS = non-significant; data are pooled means of 2017–2018 and 2018–2019 cropping seasons.

### Soil microbial parameters

Soil alkaline phosphatase activity, acid phosphatase activity and soil microbial biomass carbon (SMBC) varied significantly (p < 0.05) under different NPK treatments (Table 2). There was no significant difference (p > 0.05) of different NPK fertilizer treatments on urease activity. Under the RDF3 treatment, soil alkaline phosphatase (14.31 μg PNP g soil^−1^ h^−1^), acid phosphatase activity (4.60 μg PNP g soil^−1^ h^−1^) and SMBC (524 μg g soil^−1^) were highest, with lower levels of soil alkaline phosphatase activity (13.74 and 13.44 μg PNP g soil^−1^ h^−1^), acid phosphatase activity (4.54 and 4.14 μg PNP g soil^−1^ h^−1^) and SMBC (509.8 and 465.9 μg g soil^−1^) in the RDF2 and RDF1 treatments, respectively. The lowest enzyme activity and SMBC occurred in the RDF1 treatment.

**Table 2:** Effect of diverse fertility scenarios and multi-micronutrient foliar fertilization on soil microbial activities of eggplant.

Treatment	Dehydrogenase (μg TPF g soil^−1^ day^−1^)	Alkaline phosphatase (μg PNP g soil^−1^ h^−1^)	Acid phosphatase (μg PNP g soil^−1^ h^−1^)	Urease (µmole ammonia g^−1^ h^−1^)	SMBC (μg g soil^−1^)
**Diverse fertility scenario**					
RDF_1_	3.09_c_	13.44_c_	4.14_c_	17.50_a_	465.9_b_
RDF_2_	3.19_b_	13.74_b_	4.54_b_	18.7_a_	509.8_b_
RDF_3_	3.25_a_	14.31_a_	4.60_a_	17.8_a_	524.0_a_
LSD (*p* = 0.05)	0.08	0.61	0.28	NS	29.48
**Micronutrient embedded NPK (MNENPK) fertilizer**					
MNENPK_1_	2.79_d_	12.69_cd_	3.92_d_	17.2_c_	454.3_d_
MNENPK_2_	3.16_c_	13.29_bcd_	4.28_bc_	18.4_abc_	485.3_c_
MNENPK_3_	3.36_b_	14.47_abc_	4.64_abc_	18.4_ab_	516.2_b_
MNENPK_4_	3.41_a_	14.87_ab_	4.85_ab_	17.9_ab_	543.8_a_
LSD (*p* = 0.05)	0.07	0.48	0.24	1.28	18.1

Means followed by a similar lowercase letter within a column are not significantly different at p < 0.05 according to Tukey’s HSD test; data are pooled means of 2017–2018 and 2018–2019 cropping seasons.

*SMBC* soil microbial biomass carbon.

The effect of the MNENPK foliar-supplementation treatments was significant (p < 0.05) in terms of SMBC and all enzyme activity examined. Highest levels of soil dehydrogenase (3.41 μg TPF g soil^−1^ day^−1^), alkaline phosphatase (14.87 μg PNP g soil^−1^ h^−1^), acid phosphatase (4.85 μg PNP g soil^−1^ h^−1^) and SMBC (543.8 μg g soil^−1^) were recorded under the MNENPK4 treatment. Urease activity was significantly higher (18.4 µmoles ammonia g^−1^ h^−1^) in the MNENPK2 and MNENPK3 treatments compared to the MNENPK1 treatment (17.2 µmoles ammonia g^−1^ h^−1^). The lowest rates of dehydrogenase (2.79 μg TPF g^−1^ soil day^−1^), alkaline phosphatase (12.69 μg PNP g soil^−1^ h^−1^), acid phosphatase (3.92 μg PNP g soil^−1^ h^−1^), urease (17.2 µmoles ammonia g^−1^ h^−1^) and SMBC (454.3 μg g soil^−1^) were observed in treatment MNENPK1, which were significantly lower than in all other MNENPK treatments.

### Micronutrient concentrations in fruit, shoots and leaves

Micronutrient concentrations in eggplant fruit, shoots and leaves increased with greater applications of NPK fertilizer and of MNENPK foliar-supplementation (Table 3). The concentration of Cu in the RDF1 treatment was 0.44, 0.53 and 0.47 mg kg^−1^ in the fruit, shoots and leaves, respectively. This increased to 0.59, 0.75 and 0.68 mg kg^−1^, respectively, in the RDF3 treatment. Similarly, concentrations of Fe, Zn and Mn in the fruit, shoots and leaves increased with increasing fertilizer concentration from the RDF1 to the RDF3 treatment (Table 3).

**Table 3:** Effect of diverse fertility scenarios and multi-micronutrient foliar fertilization on nutrient concentrations in eggplant fruit, shoots and leaves.

Treatment	Cu (mg kg^−1^)			Fe (mg kg^−1^)			Zn (mg kg^−1^)			Mn (mg kg^−1^)			K (%)		
	Fruit	Shoot	Leaf	Fruit	Shoot	Leaf	Fruit	Shoot	Leaf	Fruit	Shoot	Leaf	Fruit	Shoot	Leaf
**Diverse fertility scenario**															
RDF_1_	0.44_c_	0.53_c_	0.47_c_	2.45_b_	3.17_b_	2.55_c_	1.13_c_	1.45_c_	1.19_b_	2.57_c_	3.20_c_	2.74_c_	0.209_c_	0.215_b_	0.213_b_
RDF_2_	0.52_b_	0.65_b_	0.60_b_	3.27_b_	3.88_b_	3.37_b_	1.46_b_	1.83_b_	1.56_a_	3.38_b_	3.89_b_	3.57_b_	0.242_b_	0.252_a_	0.251_a_
RDF_3_	0.59_a_	0.75_a_	0.68_a_	3.78_a_	4.33_a_	3.93_a_	1.64_a_	2.03_a_	1.61_a_	3.77_a_	4.33_a_	3.97_a_	0.264_a_	0.275_a_	0.262_a_
CD (*p* = *0.05*)	0.05	0.06	0.06	0.28	0.44	0.35	0.12	0.17	0.14	0.29	0.39	0.28	0.021	0.025	0.022
**Micronutrient embedded NPK (MNENPK) fertilizer**															
MNENPK_1_	0.46_c_	0.54_c_	0.48_c_	2.76_c_	3.41_c_	2.76_c_	1.16_c_	1.48_d_	1.19_c_	2.80_c_	3.43_c_	2.97_c_	0.231_a_	0.237_a_	0.234_a_
MNENPK_2_	0.50_b_	0.62_b_	0.56_b_	3.11_b_	3.71_b_	3.11_b_	1.39_b_	1.71_c_	1.43_b_	3.17_b_	3.73_b_	3.36_b_	0.246_a_	0.245_a_	0.238_a_
MNENPK_3_	0.55_a_	0.68_a_	0.63_a_	3.31_ab_	3.94_ab_	3.31_ab_	1.52_ab_	1.88_b_	1.57_a_	3.44_a_	3.95_ab_	3.64_a_	0.241_a_	0.251_a_	0.244_a_
MNENPK_4_	0.57_a_	0.73_a_	0.67_a_	3.49_a_	4.11_a_	3.49_a_	1.55_a_	2.01_a_	1.63_a_	3.55_a_	4.12_a_	3.75_a_	0.245_a_	0.257_a_	0.248_a_
CD (*p* = *0.05*)	0.03	0.05	0.04	0.21	0.25	0.21	0.09	0.11	0.09	0.21	0.24	0.21	NS	NS	NS

Means followed by a similar lowercase letter within a column are not significantly different at p < 0.05 according to Tukey’s HSD test; data are pooled means of 2017–2018 and 2018–2019 cropping season.

The MNENPK foliar-supplementation significantly improved the concentration of micronutrients in the plant parts. The lowest concentrations of Cu (0.46, 0.54 and 0.48 mg kg^−1^), Fe (2.76, 3.41 and 2.76 mg kg^−1^), Zn (1.16, 1.48 and 1.19 mg kg^−1^) and Mn (2.80, 3.43 and 2.97 mg kg^−1^) were observed in the fruits, shoots and leaves, respectively, of plants in the MNENPK1 treatment. The highest concentrations of Cu (0.57, 0.73 and 0.67 mg kg^−1^), Fe (3.49, 4.11 and 3.49 mg kg^−1^), Zn (1.55, 2.01. and 1.63 mg kg^−1^) and Mn (3.55, 4.12 and 3.75 mg kg^−1^) were observed in the fruit, shoots and leaves, respectively, of plants in the MNENPK4 treatment. There were no statistical differences in micronutrient concentrations between treatments MNENPK3 and MNENPK4.

**Figure 2:**
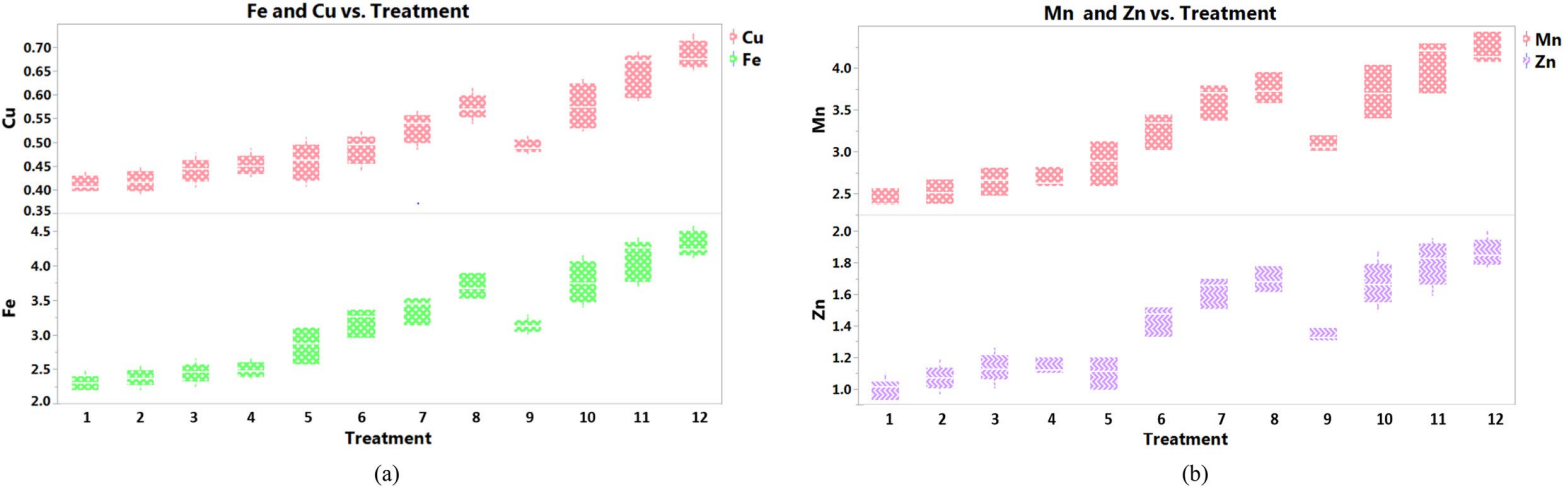
Interaction between NPK and MNENPK fertilizer treatments on eggplant fruits in terms of (**a**) Cu and Fe, (**b**) Mn and Zn content (pooled data of 2017–2018 and 2018–2019 cropping seasons). #1 = RDF1-MNENPK1; 2 = RDF1-MNENPK2; 3 = RDF1-MNENPK3; 4 = RDF1-MNENPK4; 5 = RDF2-MNENPK1; 6 = RDF2-MNENPK2; 7 = RDF2-MNENPK3; 8 = RDF2-MNENPK4; 9 = RDF3-MNENPK1; 10 = RDF3-MNENPK2; 11 = RDF3-MNENPK3; 12 = RDF3-MNENPK4.

A significant (p > 0.05) interaction effect between NPK and MNENPK treatments was observed between different micronutrients (Fig. 2a,b). The highest concentrations in eggplant fruit of Cu (0.673 mg kg^−1^), Fe (4.25 mg kg^−1^), Zn (1.83 mg kg^−1^) and Mn (4.21 mg kg^−1^) were observed in the RDF3-MNENPK4 treatment; although there was no statistical difference between this and the RDF3-MNENPK3 treatment. Increasing the application rate of the multi-nutrient fertilizer from the MNENPK1 treatment (0 kg MNENPK ha^−1^) to the MNENPK4 treatment (0.75 kg MNENPK ha^−1^), while retaining the NPK fertilizer application rate at RDF1 (0 kg NPK ha^−1^) did not result in an increase in micronutrient concentration in eggplant fruit. However, applying the MNENPK fertilizer under RDF2 (75% of RDF) and RDF3 (100% of RDF) did result in increased micronutrient concentrations in eggplant fruit.

### K concentration in fruit, shoots and leaves

The concentration of K in eggplant fruit, shoots and leaves increased significantly with increasing K under higher fertilizer applications (Table 3). The highest K content was observed in fruit (0.26%), shoots (0.28%) and leave (0.26%) under the RDF3 treatment, although there was no statistical difference between the K concentrations in shoots and leaves in the RDF2 and RDF3 treatments. The lowest K contents were observed under the RDF1 treatment (0.21, 0.22 and 0.21% for fruit, shoots and leaves, respectively), significantly lower than in the RDF2 and RDF3 treatments.

K concentration in fruit, shoots and leaves increased significantly (p > 0.05) with increasing applications of the MNENPK fertilizer, with maximum concentrations observed in the MNENPK4 treatment (Table 3). However, the RDF × MNENPK interaction effect was found non-significant for K concentration in different plant parts of eggplant.

### GGE biplot analysis

**Figure 3:**
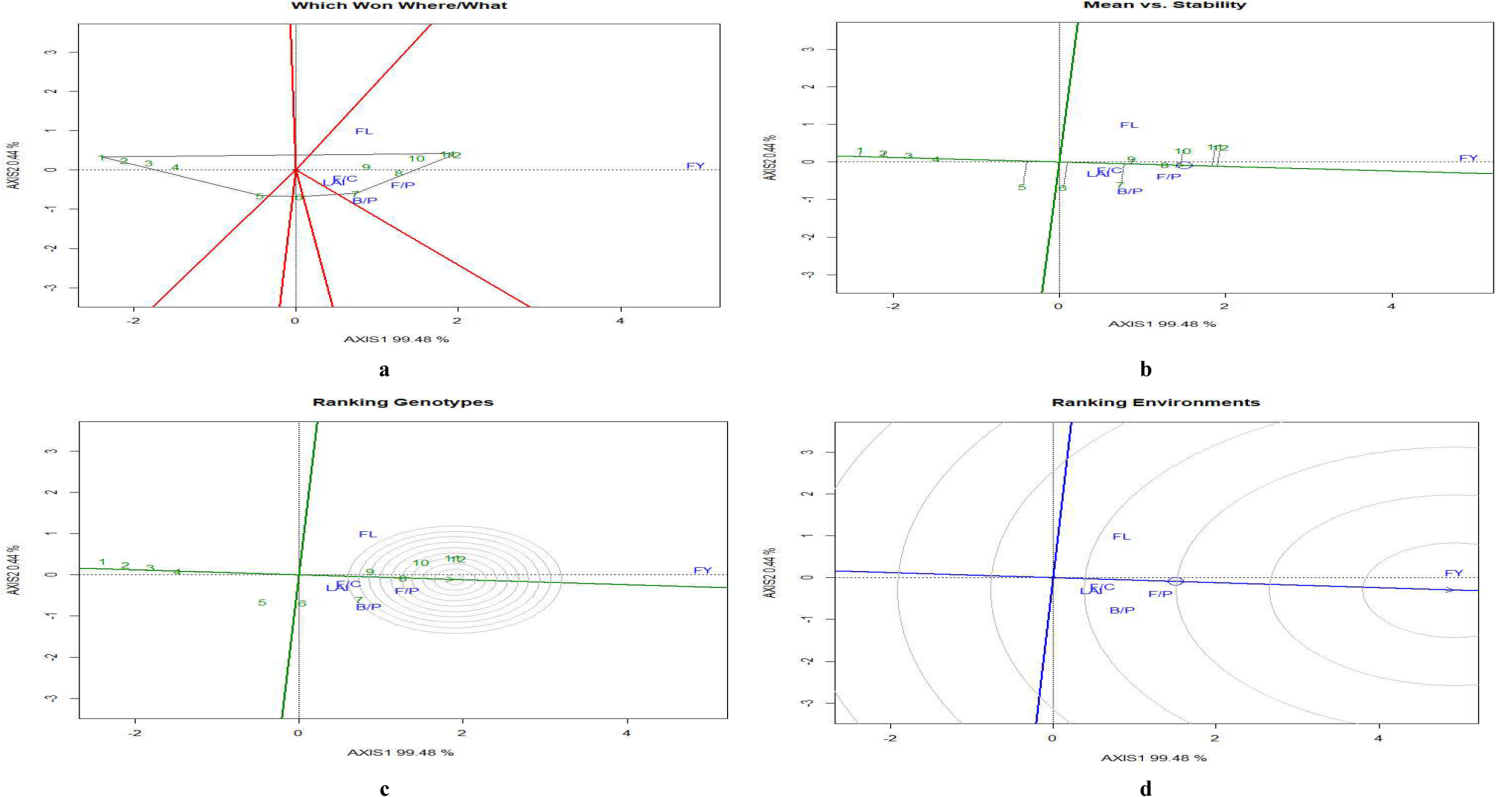
GGE biplot analysis of eggplant yield parameters under experimental treatments. (**a**) Polygon view (which won where/what), (**b**) mean vs. stability, (**c**) ranking genotypes (mean ranking treatments), (**d**) ranking environments. AXIS 1 Principal Component 1, AXIS 2 Principal Component 2. #1 = RDF1-MNENPK1; 2 = RDF1-MNENPK2; 3 = RDF1-MNENPK3; 4 = RDF1-MNENPK4; 5 = RDF2-MNENPK1; 6 = RDF2-MNENPK2; 7 = RDF2-MNENPK3; 8 = RDF2-MNENPK4; 9 = RDF3-MNENPK1; 10 = RDF3-MNENPK2; 11 = RDF3-MNENPK3; 12 = RDF3-MNENPK4.

GGE bioplot analysis was undertaken on concentrations of the micronutrients Zn, Mn, Cu and Fe and the macronutrient K in eggplant fruit, shoots and leaves. In the GGE analysis 12 treatment-combination effects and five test environments (i.e. the four micronutrients and K) were examined under the different experimental treatments. A GGE analysis of eggplant growth traits in terms of their performance under the experimental treatments was also undertaken (Fig. 3).

#### GGE biplot for nutrient concentration in eggplant

**Figure 4:**
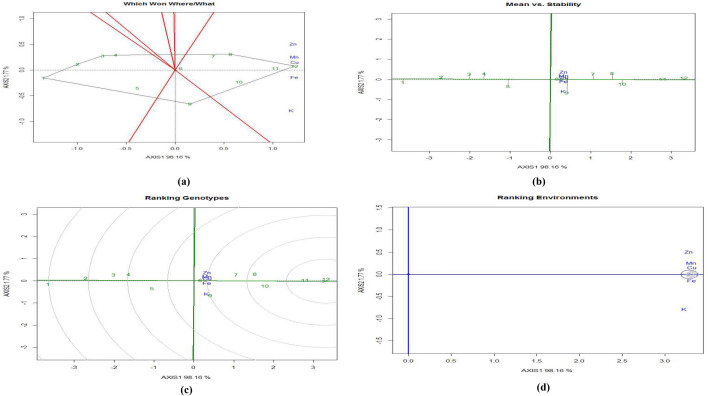
GGE biplot analysis of nutrient concentration in eggplant fruits under experimental treatments. (**a**) Polygon view (which won where/what), (**b**) mean vs. stability, (**c**) ranking genotypes (means ranking treatments), (**d**) ranking Environments. AXIS 1 Principal Component 1, AXIS 2 Principal Component 2. #1 = RDF1-MNENPK1; 2 = RDF1-MNENPK2; 3 = RDF1-MNENPK3; 4 = RDF1-MNENPK4; 5 = RDF2-MNENPK1; 6 = RDF2-MNENPK2; 7 = RDF2-MNENPK3; 8 = RDF2-MNENPK4; 9 = RDF3-MNENPK1; 10 = RDF3-MNENPK2; 11 = RDF3-MNENPK3; 12 = RDF3-MNENPK4.

In the GGE biplot analysis of micronutrients in eggplant fruit, the first two principal components (PC) explained 98.2% and 1.8% of variation, respectively. Similarly, in the GGE biplots of nutrient concentrations in eggplant shoots and leaves the first two PCs explained 97.9% and 95.8%, and 2.1% and 3.3% of variation, respectively. The ‘which won where/what’ polygon shows the two-dimensional view of multiple environments, exhibiting the best treatment across the environments and also assists in identifying the interaction pattern between treatments, years and traits (Fig. 4a). Rays divide the polygon into sectors^19^. The polygon shows that T12 (RDF3-MNENPK4) has the highest concentrations in eggplant fruit of Cu (0.67 mg kg^−1^), Fe (4.25 mg kg^−1^), Zn (1.83 mg kg^−1^) and Mn (4.21 mg kg^−1^), closely followed by T11 (RDF3-MNENPK3).

**Figure 5:**
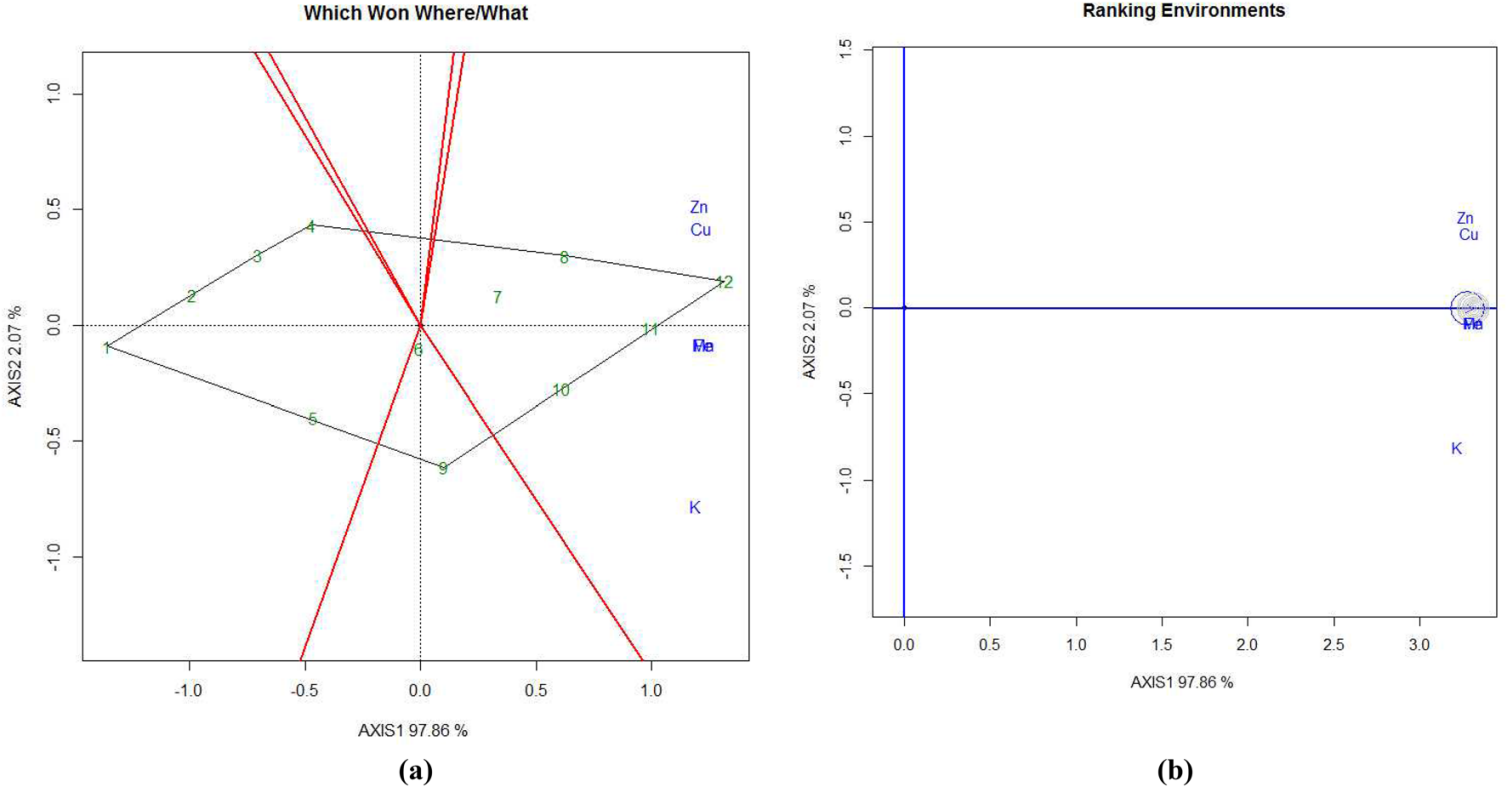
GGE biplot analysis of nutrient accumulation in eggplant shoot under experimental treatments. (**a**) Polygon view (which won where/what), (**b**) ranking environments. AXIS 1 Principal Component 1, AXIS 2 principal component 2. #1 = RDF1-MNENPK1; 2 = RDF1-MNENPK2; 3 = RDF1-MNENPK3; 4 = RDF1-MNENPK4; 5 = RDF2-MNENPK1; 6 = RDF2-MNENPK2; 7 = RDF2-MNENPK3; 8 = RDF2-MNENPK4; 9 = RDF3-MNENPK1; 10 = RDF3-MNENPK2; 11 = RDF3-MNENPK3; 12 = RDF3-MNENPK4.

**Figure 6:**
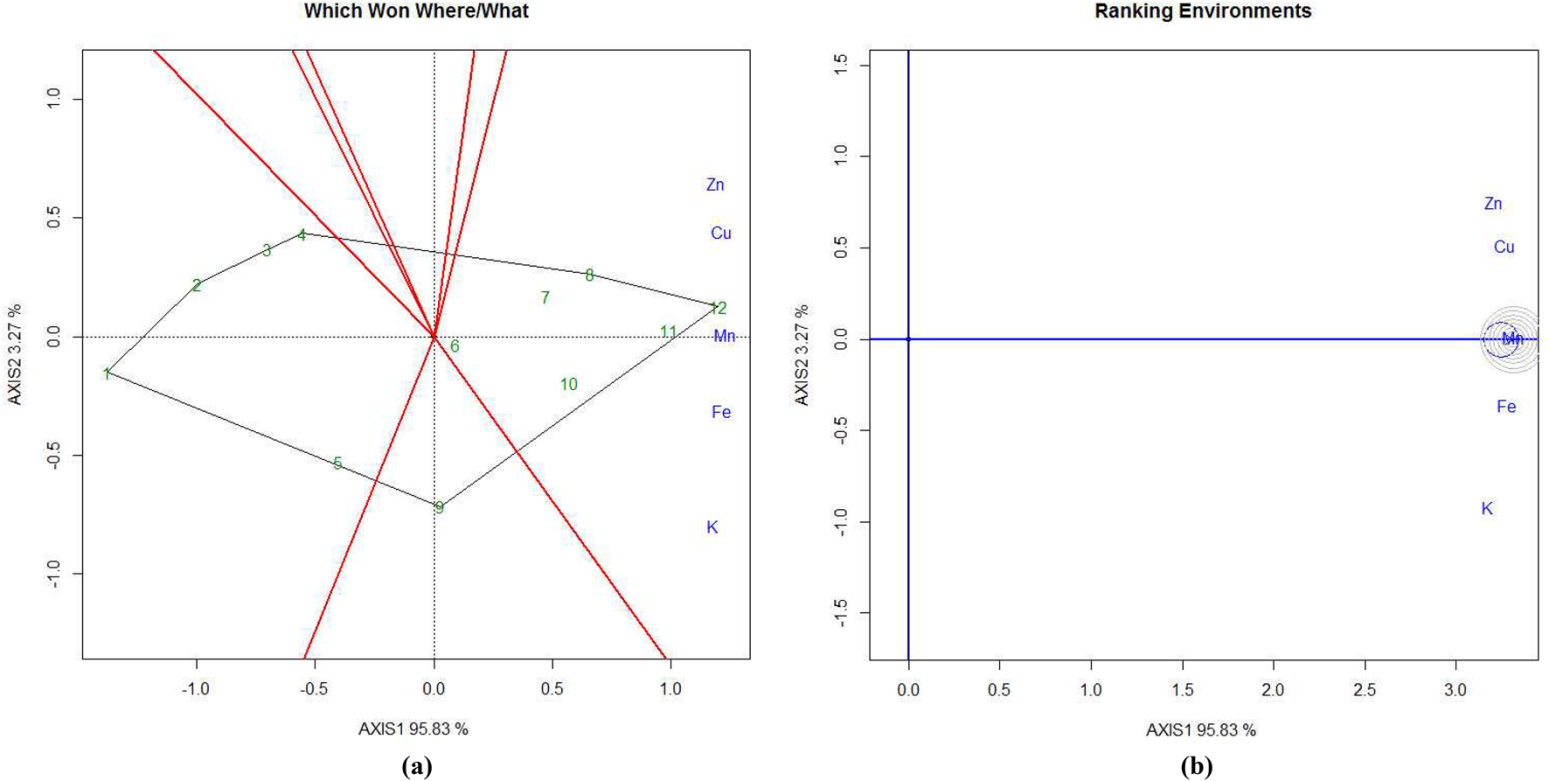
GGE biplot analysis of nutrient accumulation of eggplant leaf under experimental treatments. (**a**) Polygon view (which won where/what), (**b**) ranking environments. AXIS 1 Principal Component 1, AXIS 2 Principal Component 2. #1 = RDF1-MNENPK1; 2 = RDF1-MNENPK2; 3 = RDF1-MNENPK3; 4 = RDF1-MNENPK4; 5 = RDF2-MNENPK1; 6 = RDF2-MNENPK2; 7 = RDF2-MNENPK3; 8 = RDF2-MNENPK4; 9 = RDF3-MNENPK1; 10 = RDF3-MNENPK2; 11 = RDF3-MNENPK3; 12 = RDF3-MNENPK4.

Treatments T10, T11, and T12 are positioned in the same mega environment and are relatively close to each other, indicating that the RDF_3_-MNENPK_3_ and RDF_3_-MNENPK_4_ fertilizer applications led to the highest Cu concentration in eggplant fruits. Similar trends in nutrient concentrations were observed in shoots (Fig. 5a) and leaves (Fig. 6a).

After grouping all twelve nutrient experimental treatments into three convex hulls, all RDF1 treatments and all RDF_2_ treatments without MNENPK performed poorly in terms of nutrient biofortification and thus constituted a single mega-environment. This suggests that the application of MNENPK fertilizer alone is not sufficient to enhance the nutritional properties of eggplant fruit without RDF of NPK fertilizers. The treatment combination T9 is in a separate mega-environment which shows that in-soil application of NPK macronutrients without micronutrient supplementation does not significantly enhance micronutrient concentrations in eggplant fruit. All five micronutrient-fortified treatments in the same mega-environment indicates that, while there may be significant variations between the micronutrient treatments, there were no extreme differences in the pattern of biofortification.

#### The mean vs. stability

GGE biplots assist in identifying the treatment with the highest micronutrient concentration in fruit and the best stability. The ideal nutrient concentration and greater treatment stability are determined by a biplot’s average environment coordinate (AEC). The normal lines to the AEC which pass via through origin of the biplot are the “AEC ordinate” (Fig. 4b). In both directions of the AEC ordinate, points which are further away from the origin have lower stability and higher treatment by environment (T × E) interactions. The instability of any treatment is directly proportional to the absolute length of the projection on the AEC^20^. Treatments T10, T11, and T12 were observed to be ideal treatments in terms of nutrient concentration and stability.

#### Ranking experimental treatments

Ranking treatments within GGE biplots are used to determine the order of efficiency of the treatments (Fig. 4c). The most efficient treatment combination is one which results in the highest nutrient fortification and greatest stability across the test environments; this is placed at the centre of the concentric circles. Treatments closer to the concentric circles of the ‘ideal treatment’ have higher mean nutrient biofortifications and higher stability for nutrient accumulation. Of the various treatment combinations, the best-ranking treatments were T12 > T11 in the inner orbit, followed by T10 > T8 in the second orbit, > T7 > T9 > T6. Treatments T1 to T4 were within the two outermost circles, indicating their relatively lower performance in terms of nutrient accumulation in eggplant fruit. Figures 4d, 5b, 6b rank the nutrient concentrations relative to the ‘model environment’ depicted by the smallest circle on the AEC axis. A nutrient closer to the intersection of the straight lines has a superior ranking, whereas those farther from the intersection have lower rankings. Nutrient concentrations in eggplant fruit were ranked from Cu ≈ Fe > Mn > Zn > K, while in eggplant shoots concentrations were Mn = Fe > Cu > Zn > K (Fig. 4b). In Fig. 4b, Mn and Fe are located closer to the circles while other nutrients are further away. Nutrient concentrations in eggplant leaves were Mn > Fe > Cu > Zn > K. Except Mn, the remaining nutrients lay outside and away from the concentric circles (Fig. 5b).

#### GGE biplot for growth and yield parameters

For GGE biplot analysis of micronutrients in eggplant fruit, the first two principal components (PC) explained 99.5% and 0.5% of the variation, respectively. The ‘which won where/what’ polygon indicated that the treatments T12 (RDF3-MNENPK4) and T11 (RDF3-MNENPK3) had the highest growth and yield parameters (Fig. 3a). The presence of all growth parameters and crop yield in the same mega-environment indicates that all these parameters follow a uniform pattern of performance in these treatments. Treatments T10, T9, T8 and T7 were the second-best group of treatments in terms of crop yield and plant growth. The mean vs. stability biplots indicated that treatments T8 and T9, followed by treatments T10, T12 and T11 had the highest stability in terms of eggplant growth and yield performance. Similarly, fruit yield was the most stable trait under different treatment combinations, followed by flowers per cluster and fruits per plant (Fig. 3b). Fruit length and number of branches plant were the least stable traits. The ranking-treatments graph shows the performance order of various experimental treatments. The order for growth and yield was T12 > T11 > T10 > T8 > T9 > T7; all these treatments were in the same convex hull (Fig. 3c). Treatments T1 to T5 performed relatively poorly in terms of growth and yield. The ranking environments biplot (Fig. 3d) indicated that fruit yield was the most stable trait. The number of fruits per plant, number of branches per plant and the number of flowers per cluster were in the same orbit and this group was the second-most-stable trait group. The LAI was lying furthest from the concentric circle and was the least stable trait.

## Discussion

The supply of essential plant nutrients in optimum proportions at different growth stages is vital to increase crop yield and nutrient-use efficiency^21–23^. Optimal nutrient supply is particularly important in crops like eggplant with a heavy nutrient demand^24^. In this experiment, the balanced supply of essential NPK macronutrients supplemented by micronutrients applied as foliar spray at critical growth stages significantly improved eggplant yield and growth parameters^25,26^.

Foliar application of nutrients, especially micronutrients at later crop stages is of prime importance in enhancing the crop yields and increasing the use efficiency of micronutrients^27^. Moreover, absorption of foliar-applied nutrients is much faster than those applied into the soil^18–32^. During reproductive stages, roots are less efficient and nutrients are transported from leaves to grain or fruit^33^. Therefore, foliar feeding at fruit-development stages will supplement the amount of nutrients required to be extracted from plant leaves.

Supply of macro-nutrients like N, P and K in optimal proportion is required for proper plant growth, to reduce flower and fruit drop, and for the development of effective rooting systems which will facilitate absorption of soil nutrients^34,35^. In this research, soil applications of the RDF combined with later stage foliar fertilizing with multi-nutrient fertilizer increased the nutrient concentrations in fruits, shoots and leaves: this improved uptake of micronutrients, facilitated by well-grown plants which were a consequence of well-developed root systems resulting from ensured macronutrient supply.

Increases in concentrations of Cu and Mn in various plant parts may be a result of the development of improved source-sink channels arising from increased microbial activity in the rhizosphere (Table 2), and improved root development^34^ leading to vigorous plant growth (Fig. 1), and enhanced accumulation of these micronutrients.

Significant improvement in microbial enzyme activity and soil microbial biomass carbon (SMBC) was recorded with increasing fertilizer application. Microbes require nutrients for their growth, development and metabolism^36,37^^**.**^ Nutrient application enhances both above ground and below ground growth of plants, thereby increasing the rhizosphere area^38^ and facilitating greater microbial activity^39^. This may contribute to the increased enzyme activity and SMBC with increasing nutrient supply observed in this experiment. Improvement in soil enzyme activity was also observed by Bana et al.^37^ and Chen et al.^40^ as a result of improved crop nutrition, resulting in rhizo-deposition of nutrient-rich decayed roots and root exudates as a microbial substrate. Hartman and Richardson^41^, Pal et al.^42^ and Aeron et al.^43^ also highlighted the importance of N and P availability for microbial activity.

Plants absorb nutrients from the soil for their growth and development, which can lead to the depletion of essential plant nutrients in agricultural soils^44^. Adding essential plant nutrients via exogenous sources like fertilizers or organic manures is necessary to sustain crop yields^6^. A regular supply of nutrients at optimum levels can also improve the health and nutrient status of soils^45^. In our study, a significant enhancement in soil nutrient status was observed with the application of fertilizers. The enhancement in soil nutrient levels was observed after crop harvest due to the balanced essential nutrient supplies in plant-available forms, which also led to high rhizospheric biomass production, increasing soil organic matter and microbial activity, and ultimately improving soil fertility^46–48^.

There was no effect of the multi-nutrient fertilizer on plant-available N and K in the soil, as it was applied to plant leaves. Furthermore, the amount of the multi-nutrient fertilizer applied to the crop was too low to affect soil nutrient status. Enhanced microbial activity, specifically alkaline phosphatase, in the soil as a result of foliar nutrition may have increased plant-available P in the soil. An increase in plant-available soil P due to improved plant nutrition was also reported by Meena et al.^38^ and Pal et al.^42^ in similar soils and agro-ecologies.

## Conclusion

This research has demonstrated that foliar application of novel micronutrient-embedded NPK (MNENPK) fertilizers assists in biofortification of essential micronutrients (Fe and Zn) in eggplant fruits, which are crucial for healthy human nutrition. Application of the MNENPK fertilizer also increased the concentration and plant uptake of other micronutrients (Cu and Mn) through positive interactions, thus further improving the nutritional profile of eggplant fruits. Combined with the RDF of NPK, foliar supplementation with MNENPK is a cost-effective, sustainable strategy, which is readily accessible to farmers and will increase the yield and micronutrient concentration in eggplant, while improving soil fertility. Therefore, foliar sprays of MNENPK complex fertilizers combined with other modern crop management practices should be recommended to eggplant farmers in South Asia and other similar agro-ecologies. Further investigation into the biofortification potential of foliar fertilizers in other important vegetable crops should be a major research priority.

## Method and materials

### Experimental site

A 2-year (2017–2019) field experiment was conducted at the Division of Agronomy, ICAR–Indian Agricultural Research Institute, New Delhi (28° 4′ N, 77° 12′ E, 228.6 m altitude), on a sandy loam Inceptisol soil. Composite soil samples were taken at 0–150 mm depth before sowing, using a core sampler. Soil samples were analyzed for soil physical and chemical properties. The experimental soil had low organic carbon and plant-available N, moderate levels of plant-available P and plant-available K, and was slightly alkaline (Table 4). The plant-extractable Zn, Fe, Mn, Cu within the composite soil samples was 0.58, 4.82, 5.2 and 1.71 mg kg^−1^, respectively, before the experiment commenced.

**Table 4:** Physical and chemical properties of soil of the experimental field.

Particulars	Content	Method of analysis
**A. Soil particle size analysis**		
Sand (%)	61.6	Modified hydrometer method^49^
Silt (%)	12.6	
Clay (%)	25.8	
Soil texture class	Sandy loam	
**B. Other soil physical analysis**		
1. Field capacity (%)	18.81	Pressure plate apparatus^50^
2. Permanent wilting point (%)	6.47	Pressure membrane apparatus^50^
3. Bulk density (Mg m^−3^)	1.56	Core method^51^
4. Infiltration rate (cm h^−1^)	1.06	Double ring infiltrometer
**C. Soil chemical analysis**		
1. Organic carbon (%)	0.45	Walkley and Black method^52^
2. Available N (kg ha^−1^)	162.5	Modified Kjeldahl’s method^53^
3. Available P (kg ha^−1^)	13.9	Olsen’s method^54^
4. Available K (kg ha^−1^)	231.2	Flame photometer method^53^
5. pH (1:2.5 soil:water)	7.7	Blackman’s Xeromatic pH meter^53^
6. EC (dS m^−1^ at 25 °C)	0.35	^53^

### Treatment details

The experiment was conducted in a split-plot design replicated thrice with gross plot size of 14.6 m^2^. The recommended dose of NPK-fertilizer (RDF) for eggplant is 150 kg N ha^−1^, 26.2 kg P ha^−1^, and 49.6 kg K ha^−1^. There were three recommended dose of NPK-fertilizers (RDF) main-plot treatments viz*.*, control in which no fertilizer was applied (RDF1), 75% recommended dose of NPK-fertilizers (RDF2) and 100% recommended dose of NPK-fertilizers (RDF3). The sub-plot treatments were applications of micronutrient-embedded NPK complex fertilizer (MNENPK) as foliar spray once at flowering (full bloom stage) and another at fruiting stage (when fruits attain half size) at rates of 0, 0.25_,_ 0.5, and 0.75 kg MNENPK ha^−1^ (Table 5). The MNENPK product used in the present study contains 2.5% N, 3.91% P, 15.65% K, 0.1% Fe, 0.15% Zn, and 0.1% B, with 100% solubility in water.

**Table 5:** Details of treatments applied to eggplant.

Treatment	Treatment combination	Treatment description
**Fertility scenario 1**		
T1	RDF1-MNENPK1	Control (no fertilizer application) + Control [no foliar application of MNENPK)]
T2	RDF1-MNENPK2	Control (no fertilizer application) + Foliar application of MNENPK @0.25 kg ha^−1^ at flowering (full bloom) and fruiting (half size fruit) stages
T3	RDF1-MNENPK3	Control (no fertilizer application) + Foliar application of MNENPK @0.50 kg ha^−1^ at flowering and fruiting stages
T4	RDF1-MNENPK4	Control (no fertilizer application) + Foliar application of MNENPK @0.75 kg ha^−1^ at flowering and fruiting stages
**Fertility scenario 2**		
T5	RDF2-MNENPK1	Application of 75% of recommended dose of fertilizer (RDF) + Control [no foliar application of MNENPK)]
T6	RDF2-MNENPK2	Application of 75% RDF + Foliar application of MNENPK @0.25 kg ha^−1^ at flowering and fruiting stages
T7	RDF2-MNENPK3	Application of 75% RDF + Foliar application of MNENPK @0.50 kg ha^−1^ at flowering and fruiting stages
T8	RDF2-MNENPK4	Application 75% RDF + Foliar application of MNENPK @0.75 kg ha^−1^ at flowering and fruiting stages
**Fertility scenario 3**		
T9	RDF3-MNENPK1	Application of 100% RDF + Control [no foliar application of MNENPK)]
T10	RDF3-MNENPK2	Application of 100% RDF + Foliar application of MNENPK @0.25 kg ha^−1^ at flowering and fruiting stages
T11	RDF3-MNENPK3	Application of 100% RDF + Foliar application of MNENPK @0.50 kg ha^−1^ at flowering and fruiting stages
T12	RDF3-MNENPK4	Application of 100% RDF + Foliar application of MNENPK @0.75 kg ha^−1^ at flowering and fruiting stages

### Management of crop

Eggplant seedlings of the ‘Pusa Shyamla’ variety were grown in raised beds (7.0 × 1.5 × 0.15 m) in the second week of July in both experimental years. The seed rate was 250 g ha^−1^. In the main experimental plots, 50% of fertilizer N and 100% of fertilizer P and K were applied before the eggplant seedlings were transplanted. The remainder of the N fertilizer was applied in two equal splits, at flowering and fruit development. Seedlings were transplanted into the main field at four weeks of age, at a spacing of 0.65 × 0.65 m. A light irrigation of 45 mm depth was applied after transplanting. For weed control, pendimethalin was applied at 0.75 kg active ingredient (a.i.) ha^−1^ as pre-emergence, followed by manual weeding at 25 and 45 days after transplanting (DAT). MNENPK was applied as a foliar spray at the flowering (full bloom stage) and fruiting stage (when fruits attain half size), as per the experimental treatment plan, using a battery-powered knapsack sprayer (Table 5). For protection from fruit and shoot borer infestations, emamectin benzoate at 200 g ha^−1^ was applied during flowering and fruit formation stages. Eggplant fruits were harvested at regular intervals at horticultural maturity. The fruits used for nutrient analysis were harvested at peak fruiting stage from randomly selected plants within each experimental plot.

### Plant growth, yield and yield-attributing parameters

Key eggplant growth parameters, plant height, the number of branches per plant and the leaf area index, were recorded at the time of the third fruit harvest, from the inner rows of plots, leaving a border row on all plot sides using standard method as described by Rana et al.^55^. The yield-attributing traits (i.e. number of branches per plant, number of flowers per cluster, fruit length, and number fruits per plant) and the fruit yield were measured at horticultural maturity.

### Soil sampling and analyses of chemical status and enzymatic activity

Soil samples from 0 to 150 mm depth were collected using a core sampler to examine the effect of the treatments on soil health. Samples were taken at eggplant flowering to determine soil microbial activity and at harvest to determine soil fertility status^55^**.**

Plant-available soil N was estimated using the modified Kjeldahl method^53^. Plant-available P was determined using the Olsen method^54^**,** and plant-available K by the flame photometer method^52^. Quantification of plant-extractable Zn, Mn, Fe and Cu was done by DTPA before the commencement of the experiment^52^. To estimate topsoil microbial enzyme activity, samples were analyzed for soil microbial biomass carbon (SMBC)^56^**,** dehydrogenase^57^, alkaline phosphatases^58^, acid phosphatases^59^ and urease activities^60^.

### Estimating nutrient concentrations in plant parts

Eggplant leaves, shoots and fruits were dried, ground and digested to determine the concentrations K and of four key micronutrients, Zn, Fe, Mn, and Cu. The K concentrations were determined using a flame photometer and compared with standards ranging from 0 to 100 parts per million (ppm) of potassium chloride. Zn, Fe, Mn and Cu concentrations were estimated using an atomic absorption spectrophotometer^55^. The most sensitive wavelengths for Zn, Fe, Mn and Cu were 213.7 nm, 248.7 nm, 279.5 nm and 324.6 nm, respectively. The concentration of these mineral nutrients in different parts of the eggplant depends on genotypes or varieties. According to the USDA report 11209 (https://fdc.nal.usda.gov/fdc-app.html#/food-details/169228/nutrients; accessed on January 19, 2022), the mean values of K, Cu, Fe, Zn and Mn in eggplant fruits are 229.0, 0.090, 0.23, 0.16 and 0.25 mg 100 g^−1^ fresh weight.

### Data analyses

Means from two years and three replications in each treatment were compared using the least significant difference (LSD) test at a 95% confidence interval (Table 6). Analyses of variance (ANOVA) were conducted using SAS software, version 9.4.

**Table 6:** Analysis of variance of growth, yield attributes, yield and nutrient concentration in eggplant.

Source	DF	BPP	FL	FY	FPP	LAI	NFC	PH	Micronutrient content			
									Cu	Fe	Mn	Zn
		MS	MS	MS	MS	MS	MS	MS	MS	MS	MS	MS
Year	1	2.56	0.76	41.79	69.70	0.02	0.02	40.50	0.00095	0.00001	0.00001	0.00031
Rep (year)	4	0.07	4.59	3.63	2.40	0.47	0.56	3.47	0.00012	0.0051	0.0052	0.00089
A	2	67.79	45.42	1439.04	131.03	21.45	24.08	649.76	0.1727	12.3678	8.9126	2.1032
Year*A	2	2.83	2.61	0.40	21.42	0.14	0.07	2.93	0.0018	0.06	0.00001	0.0327
A*rep (year)	8	0.09	1.25	4.25	1.26	0.04	0.06	2.30	0.0015	0.0606	0.0609	0.0115
B	3	3.06	2.09	60.86	8.04	1.40	1.61	39.15	0.0474	1.7822	2.0062	0.6297
Year*B	3	0.15	0.62	2.07	2.34	0.01	0.02	0.20	0.00001	0.00001	0.00001	0.0025
B*rep (year)	12	0.17	0.58	5.02	0.57	0.04	0.02	4.98	0.0006	0.0248	0.0247	0.0047
A*B	6	0.19	0.20	2.00	0.76	0.06	0.14	0.91	0.007	0.3020	0.2451	0.0699
Year*A*B	6	0.07	0.26	0.41	0.68	0.02	0.01	1.33	0.00004	0.00001	0.00001	0.0027
Error	24	0.09	0.82	2.49	0.62	0.01	0.02	6.72	0.0014	0.0536	0.0557	0.0105

*Source source of variation, *Rep* replication, *A* main plot (NPK treatment), *B* sub-plot (MNENPK treatment), *MS* mean square, *DF* degree of freedom, *BPP* branches per plant, *FL* fruit length, *FY* fruit yield, *FPP* fruits per plant, *LAI* leaf area index, *NFC* number of fruits per cluster, *PH* plant height.

A genotype main effect plus genotype by environment interactions (GGE) biplot analysis was conducted using R to determine the effects of treatments (T) and the interaction effects of treatments × environments (T × E) of the main-plot treatments and MNENPK sub-treatments, following the approach of Yan et al*.*^61^ and Yan and Kang^62^. In the GGE polygons, four patters have been present viz*.* ‘which won where/what’, ‘mean vs. stability’, ‘ranking genotypes’ and ‘ranking environments’. The first pattern assists to identify best performing treatment across the environments and the interaction pattern between treatments, environments and characters. Likewise, to understand the relative stability of treatments across diverse environments, ‘mean vs. stability’ biplots are strong statistical tool. The ‘ranking genotypes’ and ‘ranking environments’ patterns are simple two-directional graphs which arranges the treatments and environments, respectively, in their order of efficiency in different mega-environments or sub-groups^19^. The first two PC generated from subjecting the singular-value decomposition to the data were used to construct two-dimensional GGE biplots. The data were centred on the applied NPK fertilizer (i.e. main-plot treatments) while comparing between MNENPK treatments, and centred on the applied MNENPK fertilizer (i.e. sub-plot treatments) when comparing between NPK fertilizer treatments. Symmetric scaling (f = 0.5) was used for the “which won where/what” pattern. The angles between environmental vectors defined the correlations^63,64^.

The following statistical GGE biplot model was used for data analyses:

$${\mathrm{Y}}_{\mathrm{ij}}-{\mathrm{B}}_{\mathrm{j}}=\sum_{\mathrm{k}=1}^{\mathrm{t}}{\uplambda }_{\mathrm{k}}{\mathrm{\alpha }}_{\mathrm{ik}}{\updelta }_{\mathrm{jk}} + {\upvarepsilon }_{\mathrm{ij}}$$

where Y_ij_ is the nutrient fortification in the fruit/leaf/shoot with treatment effect i (i = 1,…, n) in environment j (j = 1, …, p), and B_j_ is the mean of nutrient fortification in the j^th^ environment. The Y_ij_ data matrix was decomposed into *k* principal components (PC) (1 to t with t ≤ min (p, n − 1). The λ (1,…, t) are the singular values for the respective PC with λ1 ≥ λ2⋯ ≥ λt ≥ 0; α_ik_ (k = 1,…, t) are the eigenvectors for PC_1_, PC_2_, …, PC_t_, respectively, for each entry *i*; δ_jk_ are the eigenvectors for PC_1_, PC_2_,…, PC_t_, respectively, for each tester *j*, and ε_ij_ is the residual of the model.

#### Policy and plant use guidelines

The authors confirm that the eggplant variety (Pusa Shymla) used in the present study was a released variety which is under wide cultivation and was in accordance to international, national, and/or institutional guidelines.

#### Statement of permission to use specimens of Endangered Species

The authors confirm that no any collection of plant or seed specimens was practiced in the present study. The present research does not involve any species at risk of extinction and the convention on the trade in endangered species of wild fauna and flora.
